# Effects of early and intensive neuro-rehabilitative treatment on muscle synergies in acute post-stroke patients: a pilot study

**DOI:** 10.1186/1743-0003-10-103

**Published:** 2013-10-05

**Authors:** Peppino Tropea, Vito Monaco, Martina Coscia, Federico Posteraro, Silvestro Micera

**Affiliations:** 1 The BioRobotics Institute, Scuola Superiore Sant’Anna, Piazza Martiri della Libertà 33, Pisa 56127, Italy; 2 Rehabilitation Department Versilia Hospital, AUSL 12, Viareggio, Italy; 3 Bioengineering Rehabilitation Laboratory, Auxilium Vitae Rehabilitation Centre, Volterra, Italy; 4 Translational Neural Engineering Lab, Center for Neuroprosthetics and Institute of Bioengineering, School of Engineering, Ecole Polytechnique Federale de Lausanne (EPFL), Lausanne, Switzerland

**Keywords:** Stroke, Neuro-rehabilitation, Upper limb, Muscle synergies

## Abstract

**Background:**

After a stroke, patients show significant modifications of neural control of movement, such as abnormal muscle co-activation, and reduced selectivity and modulation of muscle activity. Nonetheless, results reported in literature do not allow to unequivocally explain whether and, in case, how a cerebrovascular accident affects muscle synergies underlying the control of the upper limb. These discrepancies suggest that a complete understanding of the modular re-organization of muscle activity due to a stroke is still lacking. This pilot study aimed at investigating the effects of the conjunction between the natural ongoing of the pathology and the intense robot-mediated treatment on muscle synergies of the paretic upper limb of subacute post-stroke patients.

**Methods:**

Six subacute patients, homogenous with respect to the age and the time elapsed from the trauma, and ten healthy age-matched subjects were enrolled. The protocol consisted in achieving planar movement of the upper limb while handling the end-effector of a robotic platform. Patients underwent 6 weeks long treatment while clinical scores, kinematics of the end-effector and muscle activity were recorded. Then we verified whether muscle coordination underlying the motor task was significantly affected by the cerebrovascular accident and how muscle synergies were modified along the treatment.

**Results:**

Results show that although muscle synergies in subacute stroke patients were qualitatively comparable to those of healthy subjects, those underlying the movement of the shoulder can reflect the functional deficit induced by the pathology. Moreover, the improvement of motor performance due to the treatment was achieved in conjunction with slight modifications of muscle synergies. In this regard, modifications of muscle synergies appeared to be influenced by the different recovering mechanisms across patients presumably due to the heterogeneity of lesions, sides and location of the accident.

**Conclusions:**

The results support the hypothesis that muscle synergies reflect the injury of the cerebrovascular accident and could document the effects of the functional recovery due to a suitable and customized treatment. Therefore, they open up new possibilities for the development of more effective neuro-rehabilitation protocols.

## Background

As widely described in literature, a cerebrovascular accident affects the ability of patients to effectively control their arms during complex motor tasks. Specifically, subjects who experienced a stroke usually show weakness and slowness while moving their arm, difficulty while generating and sustaining force, and delayed muscle contraction [1-3]. Moreover, a cerebrovascular accident alters the ability to selectively recruit muscle groups during upper limb related motor tasks [4-7], and involves strong torque coupling among muscles crossing elbow and shoulder joints [8-10]. Therefore, a brain damage is expected to significantly influence muscle enrolment and activity, involving an abnormal control of the arm while executing a motor task.

In order to investigate the relationship between the signals descending from damaged cortical areas and those leading the activation of muscles, some research groups have analyzed the effects of a cerebrovascular accident on “muscle synergies”. Briefly, the muscle synergies are considered as a potential strategy adopted by the Central Nervous System (CNS) to reduce the computational workload underlying the estimation of muscle activity (see Material and methods, for further details), reflect the modular organization of complex motor tasks, and seem to document the behavior of neuronal networks downstream of the neocortex [11,12]. Several experimental evidences on mammals [13,14] have corroborated this hypothesis showing that the control of the limb can be generated by combining modular roles, i.e., muscle synergies, encrypted in the neuronal networks of the spinal cord.

Due to the abnormal muscle activity of post-stroke patients and its consequent altered biomechanics, a cerebrovascular accident is supposed to significantly affect the modular organization underlying the motor task. It is therefore expected that the coordination of muscle synergies may be somehow influenced by the cerebrovascular trauma and may also reflect the severity of the impairment. Despite of this, literature provides contrasting results that do not allow to unequivocally clarify the effects of the trauma on the coordinated activity of muscle groups.

As matter of the fact, Cheung and colleagues [11] observed that the modular organization underlying muscle activity recorded during upper limb related tasks in post-stroke survivors, almost all characterized by mild severity of the impairment (i.e., Fugl-Meyer > 30/66), was very similar between affected and unaffected sides and despite differences in motor performance between arms, size and location of the cerebral lesion. Moreover, muscle synergies of patients appeared striking similar to those of a healthy control group [11]. In spite of this, the authors noticed that a cerebrovascular accident may affect the activation coefficients of muscle synergies, leading to the hypothesis that the trauma alters the cortical activation patterns for downstream muscle synergies involving motor dysfunctions in the affected arm.

Similar results were also observed by other authors [15] who observed that a cerebrovascular accident does not significantly alter the modular structure of the muscle synergies underlying stretch reflex coordination of the upper arm in post-stroke patients, but it can basically affect the recruitment patterns.

More recently, the previous research group [16] reported that motor modules underlying muscle activity of the upper limb in a larger group of patients reflect both the severity of functional impairment and the temporal distance from stroke onset. Specifically, in case of severe impairment, synergies related to the affected arm appeared as the merging of those of the unaffected one, which are assumed to be not changed by either the cerebral lesion or the elapse of time after that. On the other hand, in chronic post-stroke patients, the synergies of the affected arm appeared to be fractions of those observed in the contralateral arm. On the whole, according to these authors [11,16], the preservation, the merging and the fractioning of muscle synergies are three distinct re-adaptation strategies following a stroke which may reflect the multiple neural responses that occur after a cortical damage.

Roh and colleagues [17] similarly investigated the modular organization of muscle activity in severe chronic post-stroke patients while carrying out a 3D isometric contraction with the upper arm. Their results show that a cerebrovascular accident induces abnormal coordination of muscle activation by altering the structure of muscle synergies. Compared to earlier studies [11,15,16], the authors noticed that these systematic alterations did not reflect merging or fractioning of normal muscle synergies, but they involved further re-adaptation strategies following the trauma [17].

Other authors [18-21] also found that the fundamental modular organization of leg muscle co-excitation is qualitatively comparable between healthy and either post-stroke or spinal cord injured patients while walking. Nevertheless, they also observed that muscle synergies can be differently merged due to the severity of the stroke [18], or can reflect a variable muscle coordination across spinal cord injured patients [20,21].

According to reported results, the effect of a cerebrovascular accident on the coordinated activity of muscle groups still remains an open issue. Moreover, since almost all studies mainly aimed at investigating stroke related alterations of muscle synergies in a single experimental session, it is not possible to clarify whether the different results can be ascribed to the methodological differences and/or to the inherent inter-patients variability.

For these reasons, we designed a new preliminary study aimed at investigating whether the modifications of muscle synergies can be observed during the subacute phase (i.e., from about 1 week to 3/4 months after the trauma; see [22]) of a group of post stroke patients undergoing intense neuro-rehabilitative treatments. This period likely reflects rapid recovery of the motor performance due to the spontaneous ongoing of the pathology as well as the effects of therapeutic interventions [23]. Moreover, intense neuro-rehabilitative treatments have been demonstrated to be effective in reduce motor impairments of acute and subacute post-stroke patients [24].

According to the proposed experimental design, we wanted to test the hypothesis that the modifications of the functional performance of post-stroke patients due to combination of the natural ongoing of the pathology and the intense neuro-rehabilitative treatment are reflected in the re-organization of the modular activity underlying the control of the affected upper limb. If confirmed, our study will provide a significant evidence that muscle synergies are able to flexibly re-adapt under the influence of both intrinsic (i.e., the cerebral infarct) and extrinsic (i.e., the treatment) constraints.

## Materials and methods

### Participants

Six patients (4 males and 2 females, age 71.8 ± 5.4 years, range 66–82) were recruited during the subacute phase. All patients experienced a single unilateral stroke. Exclusion criteria were: bilateral impairment, severe sensory deficits in the limb, cognitive impairment or affective dysfunction that would have influenced the ability to comprehend task instructions or to perform experiments, physical impairments that would have impeded motor tasks, and inability to provide an informed consent. Table 1 reports a summary of features related to all patients at the begin of the experimental sessions.

**Table 1:** Summary of stroke patients recruited in this study

**Subjects ID**	**Gender**	**Age yrs**	**Days elapsed from the accident**	**Dominance**	**Paretic side**	**Stroke type**	**Lesion location**
Sub 01	M	82	37	R	L	I	Right cortical-subcortical frontal
Sub 02	F	66	29	R	L	I	Right Frontal-temporal-parietal
Sub 03	M	70	27	R	L	I	Right cortical-subcortical precentral
Sub 04	M	70	24	R	R	H	Left internal capsule
Sub 05	M	72	14	R	L	I	Right cortical-subcortical parietal
Sub 06	F	71	19	R	L	I	Right paramedian Pontis

Labels in the 2nd, 5th, 6th, and 7th columns refer to: *F*, Female; *M*, Male; *L*, Left; *R*, Right; *H*, Hemorrhagic; *I*, Ischemic.

Ten neurologically intact age matched subjects (5 men and 5 women, age 71.2 ± 5.8 years, range 64–80) were involved in the study as control group. Healthy participants exhibited normal range of motion and muscle strength, and they did not show any apparent functional disability.

All participants signed an informed consent before starting experimental sessions.

### Procedures and technical apparatus

**Figure 1:**
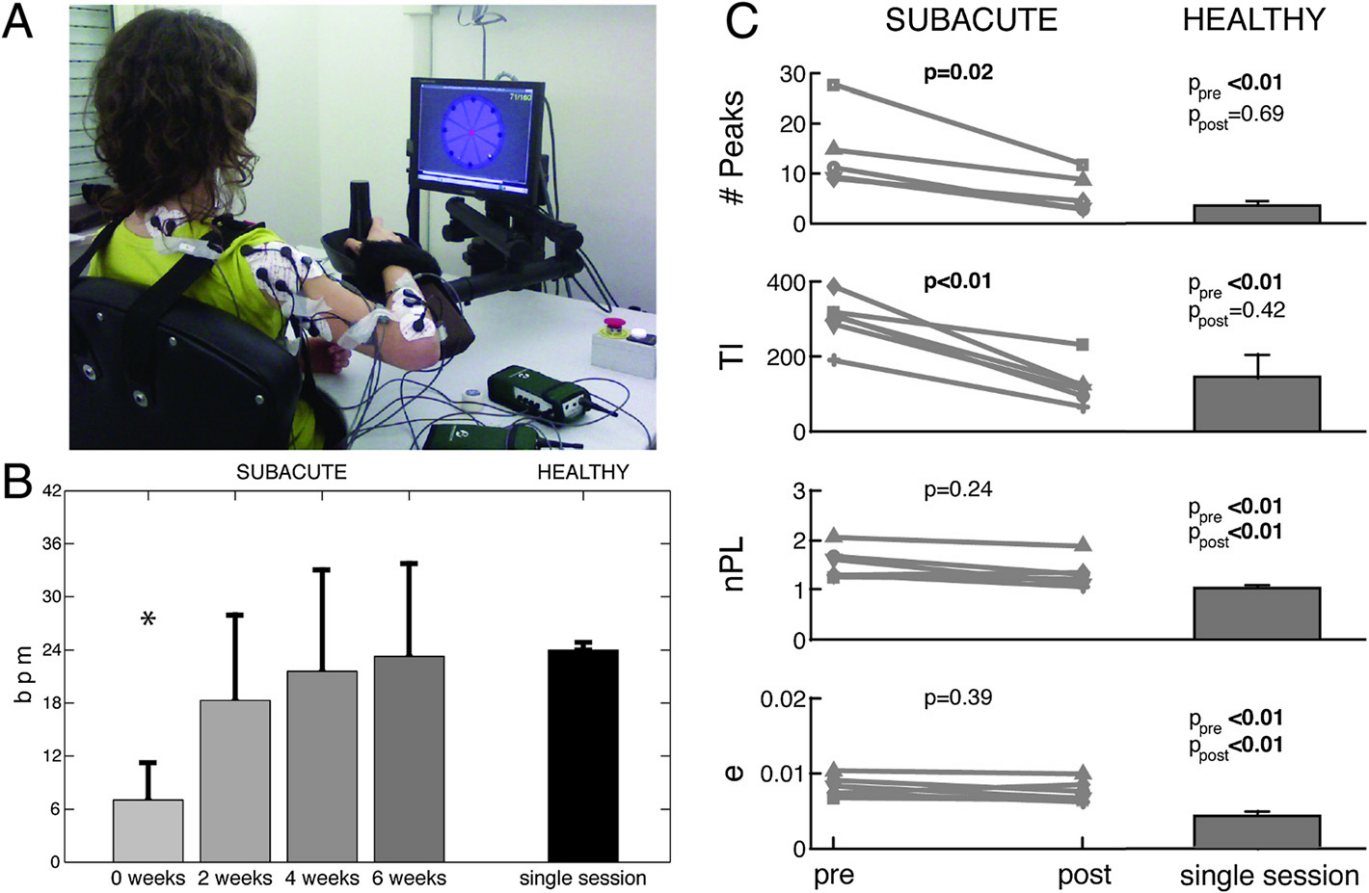
**Experimental setup, cadence of movement and robotic parameters during therapy. (A)** A subject during rehabilitative treatment. The patients moved the handle from a central target to eight ones placed around a circumference. During this task EMG signals were recorded from ten upper arm and shoulder muscles **(B)** Mean and standard deviation of cadence (bpm) in patients (four experimental sessions), and healthy participants (single session). The label * highlights when the difference between data of healthy and post-stroke patients are significantly different (p < 0.05). **(C)** Modifications of robotic parameters pre vs. post therapy. The significance of pre vs. post comparison, carried out by the Wilcoxon test (p-value), is reported above each subplot. The right column represents data related to the healthy control group. The significance (p-value) of the comparison between patients and healthy subjects is reported above each subplot. In particular: labels “pre” and “post” refer to trials before and after the treatment. P-values highlighted in bold are those statistically significant (p < 0.05).

The neuro-rehabilitative treatment, already described in literature [25], consisted in controlling the position of the end-effector of a planar robot by means of the paretic limb, while taking it forward and backward from a central target to eight ones placed around a circumference with a radius of 0.14 m (Figure 1A). When the subjects carried out all the 16 subsequent sub-movements, they completed one full turn.

The robot adopted for this study was the InMotion2 (Interactive Motion Technologies, Inc. Cambridge, Massachusetts), a platform designed to enable subjects to accomplish reaching tasks in the horizontal plane by combining elbow and shoulder angular movements [26]. In order to allow comfortable positioning, before starting the treatment, participants were asked to check if they were able to move the arm through their own full range of movement. The robot was also provided with a forearm support to compensate for the action of the gravity. During the trials, the trajectory of end-effector was recorded by the robot (sample rate at 200 Hz), and a visual feedback of the ongoing exercise was provided to each subject. Each patient received 45 minutes of robot-mediated therapy, five days per week, for six weeks, completing, at least, 65 turns per sessions. The physical therapist instructed the participants to move the handle from one target to another one while keeping the trajectory as straight as possible. Assisting force was provided by the robot when subjects were not able to reach specified targets.

Starting from the first day and every two weeks, patients attended an additional session where they carried out one further full turn, without providing any assistance. During this session, EMG signals were recorded from ten upper arm and shoulder muscles: Biceps, BIC; brachial, BRAC; brachioradialis, BRAD; anterior deltoid, DEL_A_; medial deltoid, DEL_M_; posterior deltoid, DEL_P_; latissimus dorsi, LAT; pectoralis major, PEC_M_; upper trapezius, TRAP; and triceps, TRI. Dual Ag–AgCl snap electrodes with an inter-electrode spacing of 2 cm were used during the experiments. A standard procedure, in accordance with surface electromyography for non-invasive assessment of muscles (SENIAM) guidelines [27], was used for skin preparation and electrode placement. The reference electrode was placed over the electrically neutral lateral epicondyle where it interfered least with the movement and other electrode sites. EMG electrodes were connected to a hub and wirelessly transmitted to the Noraxon data acquisition system (NORAXON, Telemyo 2400T, V2), to enable unimpeded movements. Sample rate was set at 1500 Hz.

Healthy subjects underwent a protocol similar to the one used for post-stroke patients consisting of five experimental sessions, at different cadences, composed of a 10 minute long warm-up period and 5 full turns. Only data related to the first turn were used for further analysis. Trials were carried out without using robotic assistance or resistance but constrained by the beat of a metronome at the following frequencies: 24, 30, 40, 60, and 80 beats per minute (bpm). Then, only data more closely related to the cadence of patients at discharge were used for further analysis.

The protocol was approved by the Local Ethical Committee.

### Data analysis

#### Clinical assessment

The severity of the impairment of patients was evaluated using clinical scales provided by an experienced physiatrist. In particular, muscle spasticity was quantified with the Modified Ashworth Scale (MAS), by rating resistance to passive stretch [28]. The sensorimotor status of each patient was evaluated using the upper limb section of the Fugl-Meyer Assessment (FMA) scale including items assessing upper extremity motion, balance, sensation and range of movement [29,30]. The upper limb component of the Motricity Index (Motricity) was then used to grade motor activity in muscles of the upper limb [31]. For each subject, the clinical assessment was performed at the admission, in the middle, and at the discharge of the rehabilitative treatment.

#### Analysis of the end-effector trajectory

The end-effector trajectory was low-pass filtered (zero-lag Butterworth, 4th order, cut off at 10 Hz) and analyzed to monitor the effectiveness of the ongoing therapy. For this purpose, the following metrics, related to the whole turn, were computed:

– number of Peaks (#Peaks) of the speed profile; if a point-to-point reaching movement has a low number of peaks, it means that few acceleration and deceleration periods are present [32];

– smoothness described by the Teulings’s index (TI) is the rate of change of the acceleration in a movement [33]; a lower value of TI indicates a smoother movement;

– movement accuracy was evaluated using the Normalized Path Length parameter (nPL), as described by Colombo and colleagues [34]; it virtually is the line obtained by normalizing the effective length path with the ideal one; when this parameter approximates one, movement accuracy is very high;

– the absolute hand path error (e), as computed by Franklin and colleagues [35], is the area between the actual movement path and the straight line; this was considered an index of learning and a reduction of e indicates a better adaptation to the required task.

#### Extraction of muscle synergies

Before extracting muscle synergies, raw EMG signals were pre-processed in accordance with previous literature [11]. Specifically, signals firstly underwent the cascade of high-pass-filtering (FIR filter, 50th order, cutoff at 50 Hz), rectification, low-pass-filtering (FIR filter, 50th order, cutoff at 20 Hz) and were finally integrated over 25 ms intervals. Then, filtered EMG data related to the complete turn were normalized to have zero mean and unit variance, and post-processed for synergy extraction.

The extraction of muscle synergies basically consists in decomposing a set of pre-processed EMG signals as a linear combination of basic temporal components [12], in accordance with the following equation:

(1) $$\underset{M\times t}{E}=\underset{M\times N}{W}\cdot \underset{N\times t}{C}+\;\mathrm{RES}$$

where:

 – $\underset{M\times t}{E}$ is the matrix of pre-processed EMG signals related to *M* muscles recorded along a *t*-long time window;

– $\underset{N\times t}{C}$ is the matrix of the *N* (with *N < M*) basic temporal components, also named activation coefficients;

– $\underset{M\times N}{W}$ is the matrix of the weight coefficients representing the algebraic transformation between the temporal components and the EMG signals; it is referred as the matrix of muscle synergies since it highlights which are the muscles working together and functionally activated by a specific temporal component;

– *RES* is a residual term that is supposed to account for noise related information.

As well known, several algorithms, based on different hypothesis, can be used to extract muscle synergies (see [36] for an exhaustive review). In this study we adopted the Factor Analysis (FA) with “varimax” rotation, which has been shown to be one among those best performing [36].

According to the equation 1), *N* should be chosen as the minimum number of muscle synergies able to capture the structural variation of the dataset, that is, each further synergy will only add noise. In order to only retain significant synergies, we adopted two alternative criteria:

– the *eigenvalue > 1* criterion; this criterion is based on the assumption that a factor can be considered significant if its explained variance is at least as much as that of one original variable [37-39];

– the number of synergies at which the slope of the cumulative variance drops below the 75% of the slope related to the shuffled dataset [11]; in particular, after shuffling, the dataset loses its intermuscular relationship and the cumulative variance of synergies extracted form it increases with almost constant slope, that is, all synergies account for the same amount of data information; this slope therefore refers to a structureless matrix and has been adopted as a reasonable threshold to identify synergies which informativeness can be considered negligible [11]; for each subject and each record, pre-processed EMG matrices have been shuffled 100 times and the 75% of the average slope across all runs was used as a threshold.

#### Statistical analysis

Since data were not distributed in a Gaussian fashion, only non-parametric statistic tests were used. More in detail, in order to verify whether clinical scores were characterized by a significant trend throughout the rehabilitative treatment, the Spearman correlation coefficient (ρ) was calculated with respect to the three clinical assessment sessions (i.e., Admission, Middle, and Discharge). As regard robotic parameters, for each subject, the median values over the first and the last four days were considered as representative of his/her admission and discharge status. Then, the Wilcoxon test was used to verify whether the patient’s performance was significantly affected by the treatment. Moreover, robotic parameters were also compared between healthy and impaired subjects, at the most similar cadence, by using the Wilcoxon test, to verify the degree of improvement of the kinematic patterns of the end-effector with respect to those of healthy subjects.

Before comparing muscle synergies and activation coefficients, they were firstly grouped across subjects using the best-matching scalar product of weight coefficients normalized to the Euclidean norm, according to previous authors [40,41]. Herein we will only refer to ordered synergies. Then, the scalar product of the weight coefficient vectors normalized to their Euclidean norm (dot) of two homologous muscle synergies was adopted to define a synthetic measure of their degree of similarity, More in detail:

– the median value of all dots calculated between weighting coefficient related synergies of coupled subjects within each group provided the intra-group similarity (dot_intra_);

– the median value of all dots obtained comparing homologous synergies of a patient and all healthy subjects were adopted to characterized the degree of similarity between that patient and the healthy control group; then, the median value across all patients was considered representative of the degree of similarity between patients and healthy subjects (dot_inter_).

Before comparing temporal activation components, they were firstly interpolated over 16000 points (i.e., data related to each of the 16 submovements were interpolated over 1000 points) in order to have datasets with the same length. Then, the activation coefficients for each of the 8 directions (i.e., N, NE, E, SE, S, SW, W, and NW) related to left hemiparetics were mirrored in order to correctly achieve the comparisons between groups. After that, the Pearson correlation coefficient (r) was used to compare the temporal components related to two homologous muscle synergies for each of the 8 directions of the motor task. In accordance with the approach adopted for the weight coefficients, r_intra_ refers to the comparison within each group and r_inter_ describes the degree of similarity of the activation between patients and healthy subjects.

Then, the trend of dot_intra_, dot_inter_ throughout the rehabilitative treatment (i.e., 0, 2, 4, and 6 weeks) was analyzed by using the Spearman’s correlation coefficient. The comparison between dot_intra_/dot_inter_ related to patients and dot_intra_ related to the healthy control group has been achieved by using the Wilcoxon test.

Data were processed by using custom routines developed under Matlab environment (Mathworks Inc., Natick, MA, USA). For all statistical tests, the significance was set at α = 0.05.

## Results

### Clinical assessment

Five patients were characterized by left hemiparesis; the remaining one was affected by right hemiparesis. For all patients the onset of the trauma occurred on average 25 ± 8 days prior to the experimental session (Table 1).

The robotic-aided therapy led to a reduction of the impairment of the hemiparetic limb in all patients, as shown by the trend of clinical scores throughout the therapy (see ρ in Table 2). In particular, the degree of improvement in accuracy and voluntary isolated movement was significantly documented by an averaged increment of the FMA score of 72.8% (range 33.3-147.4 %) between admission and discharge across all patients (Table 2). Moreover, patients were also characterized by a positive variation of the Motricity index (Table 2).

**Table 2:** Comparison in clinical outcomes during rehabilitation

	**MAS shoulder**				**MAS elbow**				**FMA**				**Motricity**			
	**A**	**M**	**D**	**ρ**	**A**	**M**	**D**	**ρ**	**A**	**M**	**D**	**ρ**	**A**	**M**	**D**	**ρ**
				**(p-Value)**				**(p-Value)**				**(p-Value)**				**(p-Value)**
Sub 01	0	0	0		0	1	1		21	33	41		40	64	67	
Sub 02	2	0	0		3	3	2		8	12	14		50	55	60	
Sub 03	0	0	0	-0.344	0	0	0	-0.131	21	29	28	0.319	51	55	55	0.371
Sub 04	1	0	1	(0.113)	1	0	0	(0.419)	19	41	47	**(0.049)**	40	84	84	**(0.023)**
Sub 05	1	0	0		1	0	0		36	47	49		66	79	92	
Sub 06	0	0	0		0	1	0		18	25	27		29	40	45	

Labels refer to: *A*, Admission; *M*, Middle; *D*, Discharge; ρ – Spearman correlation coefficient.

Text in bold highlights the statistically significant correlation coefficients.

Analysis of the MAS score concerning both districts (i.e., shoulder and elbow) did not reveal significant trends, even though ρ suggested that spasticity tended to decrease with the treatment.

### Analysis of the end-effector trajectory

The patients appreciably increased the cadence of their movements (Figure 1) throughout the therapy, such that at discharge they were, on average, able to carry out exercises with 23 bpm. Accordingly, data concerning post-stroke patients were compared to those of healthy subjects related to the constraining frequency of 24 bpm. The Wilcoxon test showed that only during the baseline (i.e., see “0 week” in Figure 1B) the cadence of the patients was significantly lower (p < 0.05) than that of the healthy control group.

The trend of all robotic related metrics reflected an improvement of motor performance, generally consisting of the reduced length of the path and its increased smoothness (Figure 1C). Nonetheless, the only metrics showing a significant difference between pre- and post-treatment were TI and #Peaks, which reached values comparable to those of the healthy control group during the last experimental session (Figure 1C).

### EMG signals and muscle synergies

**Figure 2:**
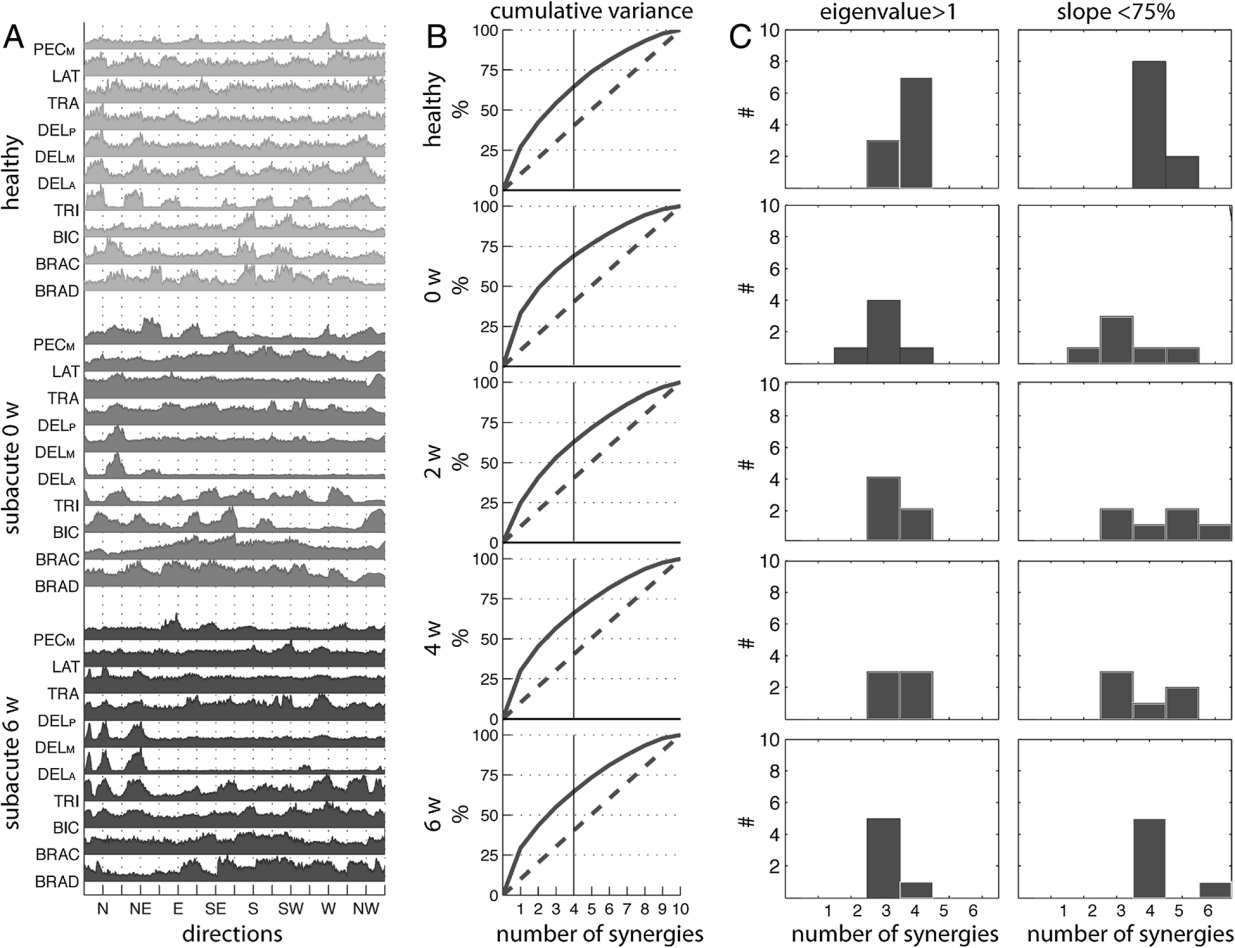
**EMG signals, cumulative variance and number of retained muscle synergies according to the adopted criteria. (A)** EMG signals related to ten upper limb muscles were collected during robot-mediated treatment. Representative EMGs for a healthy and subacute patient (at 0 and 6 weeks) are shown. Only for graphical purpose data were normalized in amplitude for their maximum value. Labels on the vertical axis are: BRAD, brachioradialis; BRAC, brachialis; BIC, biceps brachii; TRI, triceps brachii; DEL_A_, anterior part of deltoid; DEL_M_, medial part of deltoid; DEL_P_, posterior part of deltoid; LAT, latissimus dorsi; TRAP, trapezius superior; PEC_M_, pectoralis major. Labels on the horizontal axis are: N, North; NE, Northeast; E, East; SE, Southeast; S, South; SW, Southwest; W, West; NW, Northwest. **(B)** Average values of the cumulative variance related to muscle synergies extracted form the original datasets (solid line) and those extracted from shuffled dataset (dot line) as a functions of the number of extracted synergies for healthy subjects and subacute patients. **(C)** Histograms of the number of muscle synergies to be retained in accordance with the eigenvalue > 1 criterion (left column) and the slope of the cumulative variance-based criterion (right column).

EMG signals in healthy subjects were characterized by a significant modulation along the whole exercise. In particular, the visual inspection of the signals allowed to identify the anti-phase behavior of muscle groups leading elbow and shoulder flex/extension and the coordinated activity of deltoids/trapezious groups. In post-stroke patients, EMG signals were generally characterized by a higher background activity. In addition, the amplitude modulation related to some muscle groups appeared altered when compared to the healthy control group (e.g., see deltoids in Figure 2A).

Four synergies were typically considered significant to reconstruct individual muscle activation across patients and healthy subjects according to the adopted criteria (Figure 2B and 2C) even though a small subset of datasets required more modules. Despite of this, four synergies were retained from all datasets to allow an easy intra- and inter- group comparison, as already done by previous authors [17]. The cumulative variance explained by the retained synergies, in both groups, was about the 70% (Figure 2B).

**Figure 3:**
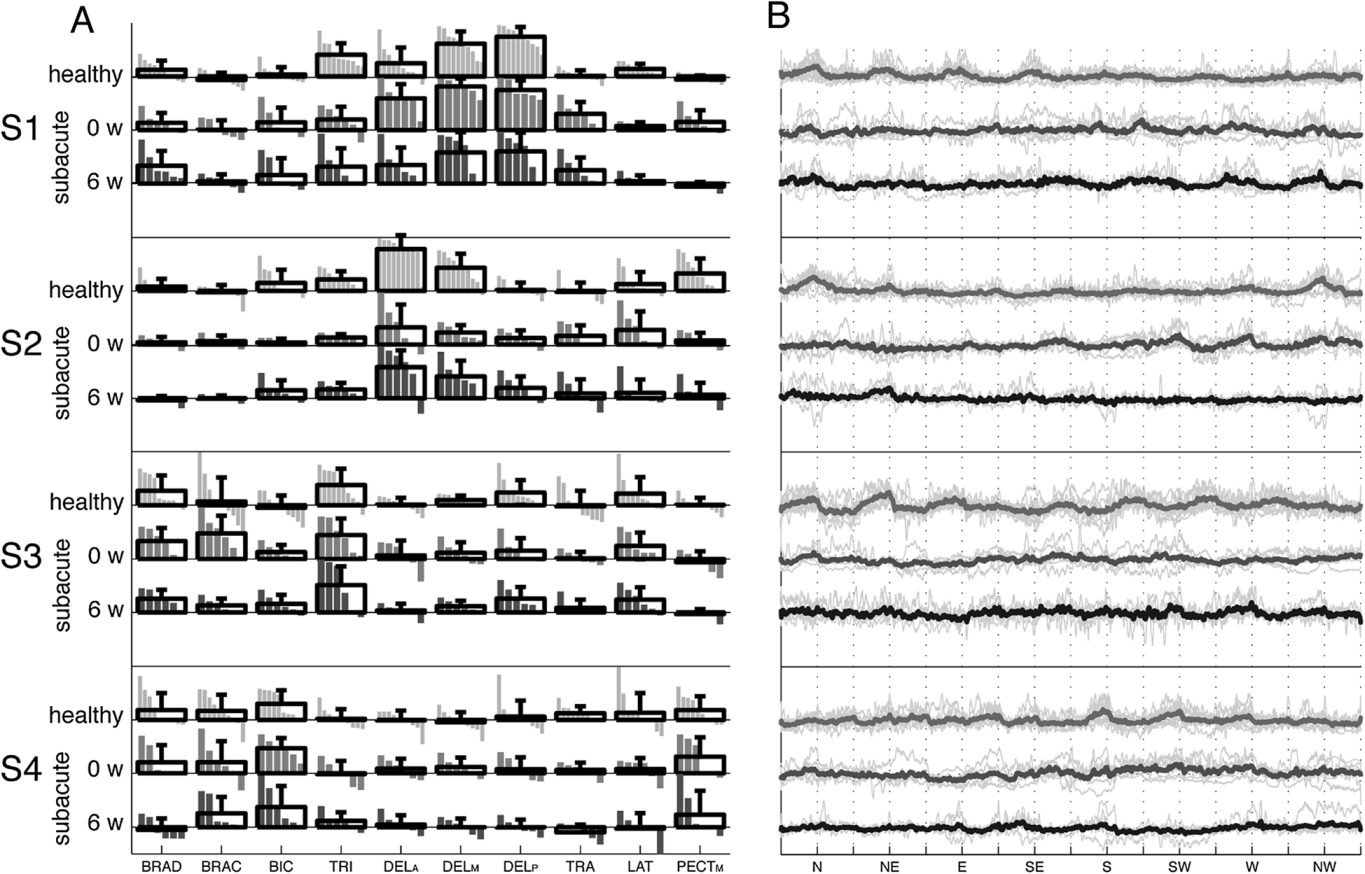
**Muscle synergies and temporal components in healthy and stroke patients.** Weight coefficients **(A)** and activation coefficient profiles **(B)** for each of extracted synergies in healthy and stroke patients before and after the treatment. Concerning the subplots **A**, gray bars show the weight coefficient for each subjects involved in the study and black bar profiles indicate group means and standard deviations. Labels on the horizontal axis are: BRAD, brachioradialis; BRAC, brachialis; BIC, biceps brachii; TRI, triceps brachii; DEL_A_, anterior part of deltoid; DEL_M_, medial part of deltoid; DEL_P_, posterior part of deltoid; LAT, latissimus dorsi; TRAP, trapezius superior; PEC_M_, pectoralis major. Labels on the horizontal axis are: 0 w, 0 weeks (pre treatment); 6 w, 6 weeks (post treatment). Concerning the subplots **B**, gray and thin lines represent the activation profiles for each individual subjects while the thick lines represent the group mean. Labels on the horizontal axis are: N, North; NE, Northeast; E, East; SE, Southeast; S, South; SW, Southwest; W, West; NW, Northwest.

Figure 3 shows both muscle synergies and temporal components underlying the coordination of muscle activity in both healthy subjects and patients. With respect to healthy control group, the first (S1) and the second (S2) synergies consisted of the activity of muscles controlling the upper arm during the abduction and the flexion/extension of the shoulder: S1 was loaded by DEL_M_ and DEL_P_, and a lower contribution of TRI and DEL_A_; S2 was loaded by DEL_A_ and PEC_M_, and the lower contribution of BIC and DEL_M_. The third synergy (S3) reflected the activity of BRAD, TRI and the lower contribution of BRAC, while controlling elbow flex/extension whereas the fourth synergy (S4) revealed the coupled coordination of elbow flexors (i.e., BRAD, BRAC and BIC) and the pectoralis major, even though it was characterized by a wide data dispersion across subjects.

Muscle synergies related to post-stroke patients (Figure 3) were qualitatively similar to those of the healthy control group even though some specific features characterized them. Specifically, S1 basically accounted for the contribution of TRI, DEL_A_, DEL_M_, and DEL_P_. Furthermore, with the ongoing of the therapy, the contribution of the TRI decreased while DEL_A_ became more consistent. S2 was mainly characterized by the activity of BRAD and BRAC. S3 also showed slightly modifications throughout the rehabilitative treatment: it initially reflected the spread activity of many muscle groups while, at the end, similar to healthy subjects, it was mainly loaded by BIC and TRI. S4 was characterized by great variability across patients which increased with the ongoing of the treatment.

**Figure 4:**
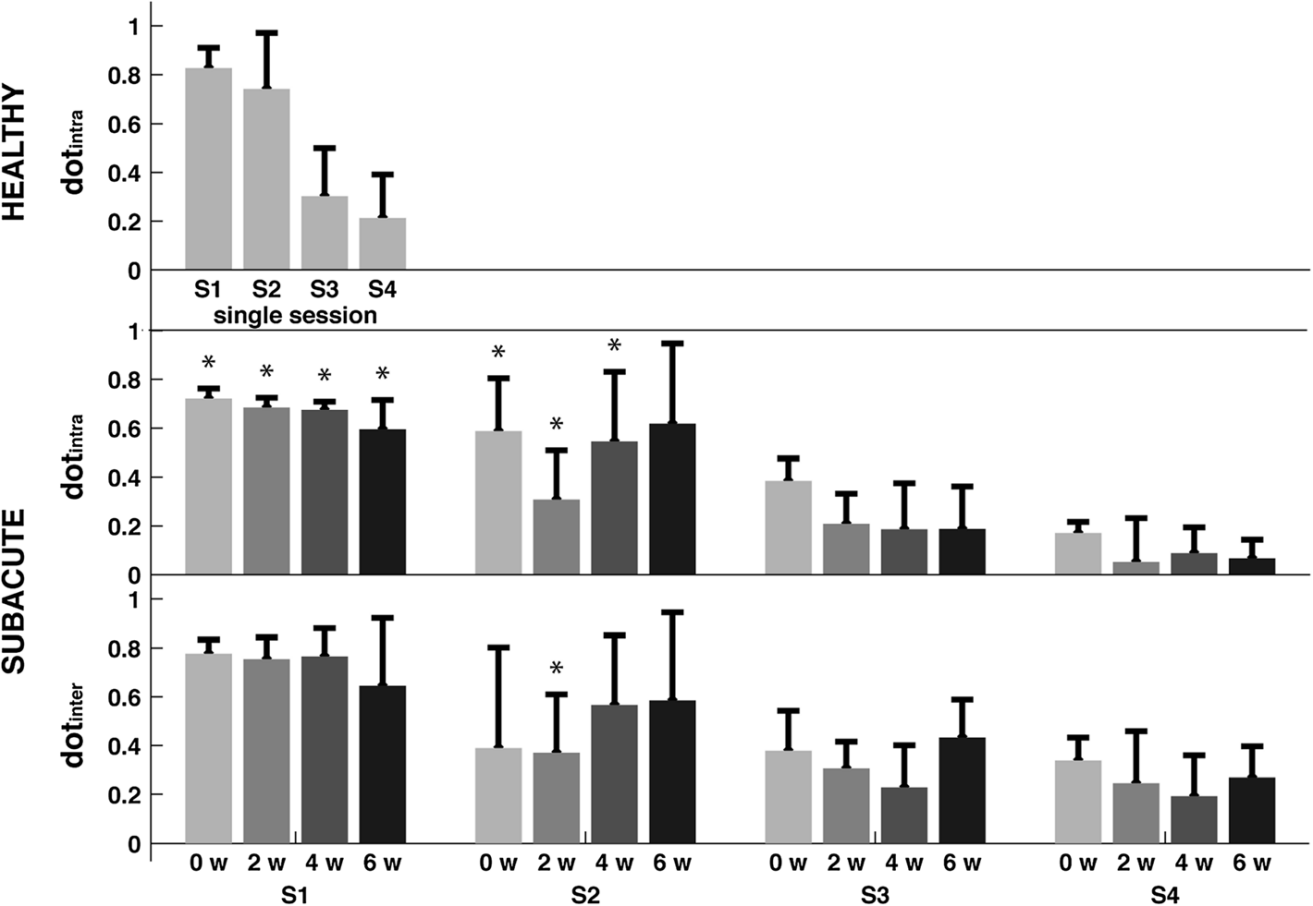
**Metrics to compare weight coefficients.** Mean and standard deviation of the metric adopted to describe intra-group (i.e., **dot**_**intra**_) and inter-groups (i.e., **dot**_**inter**_) similarity between homologous synergies. Labels on the horizontal axis refer to: 0 w, pre treatment; 2 w, 2 weeks; 4 w, 4 weeks; 6 w, post treatment. The label * highlights when values for post-stroke subjects are significantly different from healthy control group (p < 0.05).

The intra-group similarity of muscle synergies related to both healthy control group and post-stroke patients was characterized by a decreasing trend with respect to the number of retained synergies (Figure 4). For instance, in healthy subjects mean values of dot_intra_ ranged from about 0.84 in S1 to about 0.18 in S4 (Figure 4) while in post-stroke patients values were generally lower. Moreover, due to the ongoing of the treatment (i.e., at 0, 2, 4 and 6 weeks), the dot_intra_ related to patients significantly decreased for synergies S1, S2 and S4 (Table 3).

**Table 3:** **Analysis of the trend of dot**_**intra **_**and the dot**_**inter **_**along the weeks related to patients**

		**S1**	**S2**	**S3**	**S4**
**dot**_**intra**_	ρ	**-0.70**	0.26	**-0.46**	**-0.42**
	p-value	**<0.01**	0.22	**0.03**	**0.04**
**dot**_**inter**_	ρ	-0.09	0.22	0.06	-0.31
	p-value	0.67	0.29	0.78	0.14

ρ and p-value refer respectively to the Sperman’s correlation coefficient and its significance. Values in bold highlights the statistically significant correlation coefficients.

The dot_inter_ between healthy subjects and subacute patients related to S1 was significantly high (on average, about 0.76; Figure 4) whereas that related to S3, and S4 was generally characterized by averaged values lower than 0.40 (Figure 4). Differently than the other synergies, the dot_inter_ related to S2 was characterized by an increasing trend with the exception of data referring to the “0 week”, suggesting that this synergy became more similar to that of healthy subjects with the ongoing of the treatment (Figure 4). For all synergies, the Spearman coefficient highlighted that the rehabilitative treatment did not modify dot_inter_ (p-values > 0.05 in Table 3).

The temporal component related to S1 (Figure 3) in healthy subjects was characterized by well shaped peaks occurring when subjects inverted the direction of the handle (i.e., from forward to backward) which amplitude was greater during the ipsilateral movements (i.e., from the N to the SE directions) than during the contralateral ones (i.e., from the S to the NW directions). Patients did not show a consistent behavior of S1 at the beginning of the treatment. However, during the following experimental sessions, a more uniform modulation of this activation coefficient across patients appeared characterized by a greater amplitude during the ipsilateral movements of the handle.

Concerning the temporal component related to S2, in healthy subjects it was characterized by wide peaks basically during the movements toward the north-related directions. In post-stroke patients, before the treatment, the peaks of activation were consistently present during the ipsilateral movements of the handle. After the treatment, they disappeared or were characterized by a scarce consistence across patients.

The temporal component of S3, leading elbow flex-extension, showed the expected peaks along all directions in healthy subjects. Conversely, it was characterized by not uniform behavior across post-stroke patients.

The temporal component of S4 in healthy subjects showed consistent peaks across the subjects in almost all directions, even though those contralateral (i.e., S, SW and W) were characterized by the greatest amplitude. In post-stroke patients, it did not show a regular shape along the whole therapy cycle, even though after the 6 weeks of treatment it showed a better modulation.

**Figure 5:**
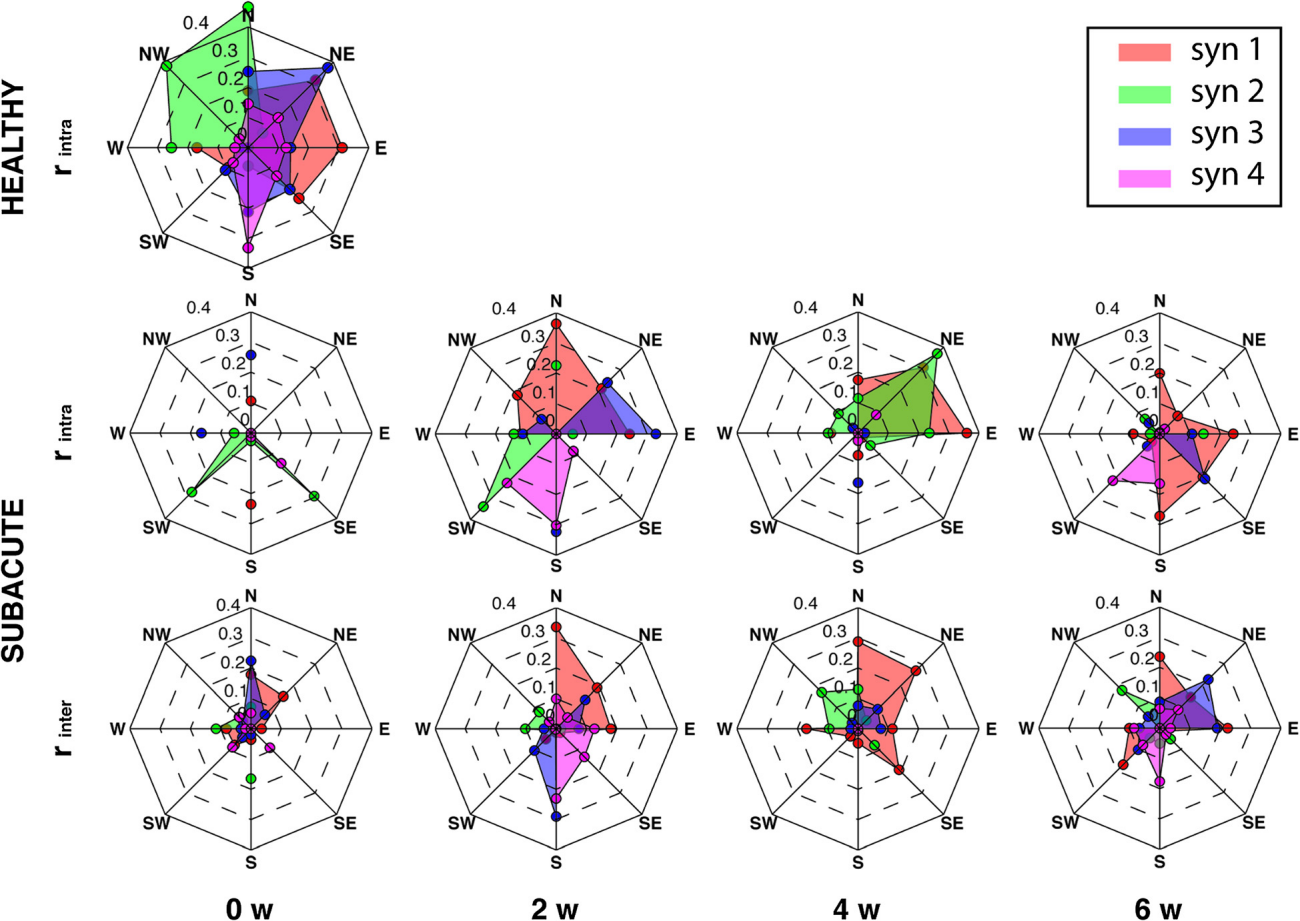
**Metrics to compare temporal components.** Mean value of the Pearson’s correlation coefficient adopted to compare intra-group (**r**_**intra**_) and inter-groups (**r**_**inter**_) similarity between homologous temporal components for each of the 8 directions. Labels on the plots are: N, North; NE, North-East; E, East; SE, South-East; S, South; SW, South-West; W, West; NW, North-West. Labels on the horizontal axis are: 0 w, pre treatment; 2 w, 2 weeks; 4 w, 4 weeks; 6 w, post treatment.

According to the visual inspection of temporal components, the intra-group similarity of activation patterns related to healthy subjects showed that each synergy was basically elicited by a set of specific directions. Specifically (Figure 5): the temporal component related to S1 was characterized by a consistent peak across subjects when the movement was directed toward NE-E-SE directions; that related to S2 was elicited by movements directed toward W-NW-N directions; those concerning S3 and S4 were more consistent when referring to all directions from N to SW, clockwise, even though, with variable value of r_intra_.

The comparison of the temporal coefficients within and between groups (Figure 5) related to post-stroke patients showed that at the beginning of the treatment they were not consistent either among patients or with the healthy subjects, and generally assumed lower values of r_intra_, and r_inter_ than the control group. With the ongoing of the treatment, both r_intra_, and r_inter_ increased suggesting that the related temporal components were elicited and became more consistent depending on the direction of the movement. Moreover, the anisotropic behavior of r_inter_ was in slight accordance with data related to the healthy subjects.

## Discussion

This study aimed at verifying whether the expected improvements in motor performance of subacute-patients due to conjunction between the spontaneous recovery and the intense neuro-rehabilitative treatment were reflected in the modular coordination of muscular activity. This pilot study was focused on a narrow sample of patients (see Materials and methods) due to the need of having a homogeneous group with respect to the time elapsed from the onset of the stroke.

As expected, muscle recruitment both in healthy and hemiparetic subjects is characterized by a certain degree of coordination, such that the activation of 10 muscles can be described by a smaller number of muscle synergies (Figure 2B), in accordance with previous studies [11,40]. Results therefore corroborate the general finding [42] that movement planning is mainly based on the organization of sub-modules which, in the framework of the adopted protocol, can be functionally related to support the arm against gravity (see S1 in Figure 3), to flex/extend the shoulder (see S2 in Figure 3), and to flex/extend the elbow (see S3 in Figure 3). Although S4 revealed the coupled coordination of elbow flexors (i.e., BRAD, BRAC and BIC) and pectoralis major, it was characterized by a wider data dispersion across subjects than previous modules.

The modular organization of roles underlying muscle activity appeared to be significantly influenced by both the cerebrovascular accident and the functional recovery. Specifically, due to the trauma, the intra-group consistence of muscle synergies (Figures 3 and 4) and temporal coefficients (Figures 3 and 5) was generally lower than in the control group. Moreover, the ongoing of the treatment involved more consistent activation patterns which appeared related to specific directions of the movement (Figure 5).

### Consistence of muscle synergies

As well known, one of the key issues of the extraction of muscle synergies consists in identifying the number of modules capturing only the systematic behavior of the original dataset. In this respect, the number of synergies at which the curve of the cumulative variance shows an abrupt change of slope is generally considered suitable for this purpose [11].

Indeed, several authors have shown that the cumulative variance grows gradually making difficult the identification of the correct number of synergies by visual inspection [11,39,41,43]. For these reasons several additional criteria have been adopted to disentangle signal from noise.

With respect to our study, we adopted 2 different criteria to identify the correct number of synergies. These methods suggested that 4 modules can be considered suitable to reconstruct the original datasets and accounted for about the 70% of the total data variance. These values are in good accordance with those reported in previous works where the authors noticed that 5-6 synergies were sufficient for explaining about the 75-80% of data variance related to 12 muscles recorded from mild-to-severe post-stroke patients while carrying out several upper limb related motor tasks [11,16].

On the whole, since the aim of our study consisted in comparing muscle synergies describing both a group of subacute post-stroke patients and a group of healthy subjects, we focused our attention on modules common to all subjects. Therefore, the number of retained synergies appeared to be well suited to describe the greatest amount of data information coded in recorded EMG signals.

Concerning the consistence of weight coefficients, results showed that only S1 in both healthy subjects and post-stroke patients was characterized by a significant degree of intra-group consistency (on average, dot_intra_ > 0.75; see Figure 4) while dot_intra_ of S2, S3, and S4 was usually lower (on average, dot_intra_ ranged between about 0.15 and 0.6). Indeed, these values do not appear in agreement with those reported in previous studies [11,40,44,45], even though this discrepancy can be reasonably explained by two main reasons concerning the experimental design and, the data processing.

As matter of the fact, there is no unanimous consensus concerning how to pre-process raw data, such that previous authors have already highlighted that different approaches can lead to different conclusions [18,39]. Moreover, synergy extraction is itself a sort of data filtering because it allows the algebraic complexity of the data set to be reduced by selecting only those factors, which are supposed to account for the greatest amount of information [41]. In this regard, by adopting different criteria to retain synergies [18,43] or to pre-process data [46], it is possible to obtain different results. Finally, other authors [47] noticed that the attitude of some factorization algorithms to accurately capture the main features of a dataset can strongly depend both on the sparseness and the diversity of the actual modules underlying the structure of the dataset being factorized, and on how uniformly the space accounting for such actual modules is parsed.

Concluding, different experimental paradigms (e.g., motor task, recorded muscle groups, age, and pathology), pre-processing and factorization (i.e., time variant versus time invariant factorizing algorithms) algorithms can have led to different degrees of similarity within homologous groups of synergies. Therefore, further efforts are needed to achieve wider agreement among the authors regarding both methods for data pre-processing and synergy comparison.

### Effects of the stroke on muscle synergies

Results (Figure 4) showed that the intra-group consistency at the baseline (i.e., “0 weeks”) of S1 and S2 of the post-stroke patients was generally lower than that of healthy subjects. This was accompanied by a very low intra-group consistency of the temporal components of all modules that was also inhomogeneous with respect to the direction of the movement (see r_intra_ in Figure 5), and by a low inter-group degree of similarity of temporal components (see r_inter_ in Figure 5). Despite of this, muscle synergies of post-stroke patients appeared similar to that of healthy subjects (see dot_inter_ in Figure 4) suggesting that they were characterized by the same modular organization.

Noticeably, these results cannot be completely ascribed to the different cadence between healthy and post-stroke patients because they were similarly confirmed when both groups carried out the motor tasks with comparable speed (Figures 4 and 5).

These evidences support previous findings concerning the robustness of muscular organization within each synergy and suggest that although the cerebrovascular accident may increase the variability between patients, the basic structure of each module is not significantly altered when compared to that of healthy subjects [11,15]. Conversely, the temporal components enabling each synergy appeared significantly altered by the trauma, indicating that they presumably result by the abnormal motor commands descending from the damaged hemisphere [11,15]. In this respect, our results corroborated the hypothesis that muscle synergies may be encrypted in neural circuits located in the spinal cord and/or in the brainstem and are inconsistently recruited due to trauma [11,17].

With respect to the specific effect of the trauma on weight coefficients related to S1 and S2, results (Figure 3) showed that patients at the baseline were mainly characterized by the abnormal contribution of deltoid heads on synergies underlying the control of the shoulder. Specifically, in post-stroke patients, the DEL_A_ significantly loaded S1 whereas, according to data referring to the healthy control group (Figure 3), it was expected to contribute to the flexion of the shoulder described by S2. Moreover, the contribution of the anterior and medial heads of the deltoid to S2 was appreciably attenuated.

Actually, previous authors [17] already noticed that, during an isometric task, the alteration of the structure of muscle synergies was mainly confined at the proximal district and affected in a negative fashion the motor performance of the patients. They hence hypothesized that this abnormal muscle recruitment in their chronic patients could result as an adaptive response to the weakness following the trauma.

Our study confirms these previous findings and reveals that the re-modulation of the contribution of the deltoid heads to the synergy leading the proximal joint may directly reflect the altered muscle recruitment early after the trauma. In this regard, it is possible to speculate that this abnormal muscle-recruitment can be considered predictive of the degree of impairment.

Actually, from the best of our knowledge, our study is the first one aimed at investigating muscle synergies of only subacute post-stroke patients. Therefore some of the results of previous studies which have been confirmed by our analysis (e.g., robustness of modules and alteration of temporal coefficients, re-modulation of the behavior of muscles crossing the proximal district) can be more directly ascribed to the effect of the trauma rather than to compensative strategies induced by the chronic stage of the pathology. However, further studies are required to stronger support to this hypothesis.

Muscle synergies related to S3 and S4 did not show significant differences between the two groups of subjects, both in term of inter- and intra-group consistence. This result is in agreement with that reported by Roh and colleagues [17] who did not notice significant alteration of muscle synergies leading the control of the distal joint. However, we cannot reject the hypothesis that it could be due to the evidence that these two synergies, together, accounted for about the 20-25% of the variance of the datasets and were therefore characterized by an intrinsically greater variability than S1 and S2. In this regard, we believe that some of the methodological issues underlying all factorization approaches should be still clarified in order to allow a confident interpretation of the analysis of muscle synergies.

### Effects of the ongoing of the treatment on muscle synergies

The conjunction between the spontaneous recovery and the intensive treatment improved motor performance, both in terms of kinematics of the end-effector (Figure 1B and 1C) and functional scores (Table 2). In particular, the therapy generally favored the reorganization of the upper limb related motor tasks and modified patient’s motor capabilities. This evidence is in accordance with previous studies, which have already highlighted that robot-assisted therapy can improve motor performance, allowing patients to learn how to coordinate their joints in adaptable patterns in order to increase the functional outcome [24,48].

Although the treatment involved functional improvements, the degree of similarity between muscle synergies of post-stroke patients and healthy participants did not significantly change with the ongoing therapy (see dot_inter_ in Figure 4 and Table 3). Conversely, the intra-group consistence of muscle synergies related to S1, S3 and S4 in patients was characterized by a slight and significantly (p < 0.05) declining trend along the treatment duration (see dot_intra_ in Figure 4 and Table 3). On the other hand, the temporal components reflected a certain degree of adaptability of the Central Nervous System (CNS) and suggested that a treatment provided early after the trauma involves the reorganization of descending signals (Figure 5). Despite of this, the activation patterns were not characterized by a stereotyped behaviour both across subjects and with the ongoing of the treatment (Figure 5).

Results therefore contrasted the hypothesis that a better motor outcome of post-stroke patients, as assessed by clinical scores and kinematics of the end-effector, was reflected in a muscle coordination more similar to that of the healthy control group and more consistent across subjects. In this regard, we believe that the reasons underlying these results reside in the complex relationship between the modifications of the motor outcome and the re-organization of muscle activity due to the treatment, and involves both methodological and neuro-physiological aspects.

From the methodological viewpoint, the improvements of motor performance due to neuro-rehabilitative therapies mainly consist of changes in amplitude modulation between agonist and antagonist muscle groups [7,10,49,50]. The FA instead captures the communality of a set of zero-scored EMG signals by means of their correlation, which mainly reflects the concomitance of signal bursts, i.e., the timing of the activity. Hence, although the FA is able to highlight the inter-muscular coordination, it may be not enough sensitive to characterize variations of the signals’ amplitude. This result is corroborated by previous authors [39,43,51] who investigated the principal roles underlying the coordination of muscle activity during walking in a wide range of speeds and reported that, despite the EMG amplitude is significantly affected by the speed, muscle synergies do not seem to be influenced by the pace of the subjects. Further methodological improvements are hence required to capture all available information encrypted in the coordinated activity of many muscle groups and, in case, highlight the trend of the dot_inter_ due to the treatment.

From the neuro-physiological side, the decreasing consistence of similarity across patients during the ongoing of the treatment (see dot_intra_ in Figure 4) could be ascribed to the different recovering mechanisms due to the heterogeneity of lesions, sides and location of the accident. In other words, the improvements of motor performance across patients occur in accordance with their own clinical picture, that is, they are characterized by great inter-subjects variability, which does not facilitate to capture univocal and common features of the whole group. In this regard, a greater number of participants can provide further evidence for this result. Moreover, from the rehabilitative viewpoint, the analysis of the correlation between motor outcome and muscle synergies should be carried out for each single patient in order to avoid bias due to the inherent inter-patients variability.

Concluding, as also observed by other authors [11,17], the analysis of muscle synergies seems to be effective in providing a theoretical support for the design of therapeutic interventions for post-stroke patients because it can highlight the neural re-organization of motor control resulting after a cerebrovascular accident and leaded by a neuro-rehabilitative treatment.

### Limits of the study

The first limit of this study was the small group of participants which involves a limited strength of the statistical findings. However, our intention was to enroll a homogenous group of patients, in term of age (range: 66-82 ys), onset from the trauma (range: 14-37 days) and absence of bilateral impairments, in order to reduce, as much as possible, the potential bias of the intrinsic lack of homogeneity across patients which may be one of the main reasons underlying the discrepant results among previous literature. Accordingly, this work has been designed as a pilot study and further investigations, according to the reported results, are guaranteed.

The second potential limit concerns the slow speed adopted by the patients before the treatment. This may bias the interpretation of the results because the value of all metrics describing kinematic parameters and muscle synergies are conjunctly affected by both the functional capabilities of all patients before starting the therapy and the slow speed achieved during the exercises. However, from one hand, the increasing speed and smoothness throughout the treatment supported the hypothesis that the therapy is effective from the clinical viewpoint. On the other hand, since during the following experimental sessions (i.e., 2, 4 and 6 weeks) patients achieved a cadence comparable to that of healthy participants (Figure 1B), our experimental design allows to extrapolate that the degree of similarity of muscle synergies between healthy subjects and post-stroke patients is characterized by a monotonic behavior.

The last limit of this study was that in only one case, a patient was affected by paresis of the dominant arm (see Sub 04 in Table 1). Actually, the effect of the interaction between dominance and affected side is widely discussed in literature even though there is not an exhaustive understanding [52,53]. With respect to our study, we did not observed any apparent difference between this patient and the others which might justify his exclusion from this study. However, we acknowledge that further studies are required to explore this issue.

## Conclusions

This pilot study supports the hypothesis that the coordinated activity of muscle groups, i.e., muscle synergies, are significantly affected by a cerebrovascular accident. Moreover, results suggest that due to the treatment, patients modify the coordinated activity of muscle groups even though the reorganization of rules underlying motor control are characterized by a significant inter-patients variability. If confirmed with a significant robustness, these insights would support the hypothesis that suitable and customized treatments can be designed to favourite the functional recovery of post-stroke patients.

## Competing interest

The authors declare that they have no competing interest.

## Authors’ contributions

PT designed and carried out experiments, analyzed data and wrote the paper; VM designed experiments, analyzed data and wrote the paper; MC carried out experiments; FP designed the study; SM designed the study, analyzed data, and wrote the paper. All authors read and approved the final manuscript.
